# Unravel a Knot: Exploring Double Edged Effects of Gratitude Mindset on Social Behaviors

**DOI:** 10.3390/bs16091628

**Published:** 2026-09-11

**Authors:** Wenya Yang, Jianwei Zhang, Guangxia Guo, Xinchen Niu, Tingting Zhang, Meiting Xiong

**Affiliations:** School of Education, Beijing Institute of Technology, Beijing 102488, China; 3120236141@bit.edu.cn (W.Y.); 3120236144@bit.edu.cn (G.G.); xinchenniu@bit.edu.cn (X.N.); tingtingzhang@bit.edu.cn (T.Z.); 3220252631@bit.edu.cn (M.X.)

**Keywords:** gratitude mindset, altruistic behavior, aggressive behavior, moral imagination, moral disengagement

## Abstract

While abundant evidence supports gratitude’s positive functions, recent findings have also revealed its potential to trigger aggressive behaviors. However, existing theories struggle to fully explain this apparent paradox. To address this gap, we introduce the construct of the gratitude mindset and explore its paradoxical impact on social behaviors. Study 1 (*N* = 221) employed an experiment to verify the distinct effect of the gratitude mindset on altruistic and aggressive behavior. Based on a three-wave survey, Study 2 (*N* = 462) revealed that a gratitude-is-enhancing mindset (GIEM) was positively associated with altruistic behavior and negatively associated with aggressive behavior, whereas a gratitude-is-inhibiting mindset (GIIM) was positively associated with aggressive behavior and negatively associated with altruistic behavior. Moreover, moral imagination partially mediated the effects of GIEM on altruistic behavior and aggressive behavior; moral disengagement partially mediated the effects of GIIM on altruistic behavior and aggressive behavior. These findings suggest that the gratitude mindset has both theoretical and practical relevance for understanding social behavior.

## 1. Introduction

Gratitude refers to a positive emotion that arises when individuals recognize that they have obtained a positive outcome and attribute that outcome to the intentional benevolence of others (McCullough et al., 2002). Historically, gratitude has been regarded as a fundamentally prosocial moral affect that motivates beneficiaries to reciprocate kindness and extend benefits to unrelated others (McCullough et al., 2001, 2002). Consistent with this view, a wealth of research has demonstrated that gratitude promotes various forms of altruistic behavior, such as helping behavior (Bono et al., 2019) and Internet-based prosocial actions (Lin, 2025). However, this uniformly positive portrait has recently been challenged by evidence that gratitude can also trigger morally questionable behaviors, including moral violations to protect benefactors (Zhu et al., 2020) and increased willingness to obey unethical commands (Tong et al., 2021). These divergent findings raise a critical question: why does the same gratitude experience lead some individuals to act altruistically, but others to act aggressively?

Existing theories have provided valuable explanations for gratitude’s functional consequences—whether as a reciprocity-driven obligation (Cropanzano & Mitchell, 2005), a moral emotion motivating prosociality (McCullough et al., 2001), or a relationship-maintenance mechanism (Algoe, 2012). Nevertheless, these accounts primarily address why gratitude produces certain outcomes at the functional level, offering limited insight into a different but equally important question: why do individuals respond to gratitude experiences in such divergent ways? We propose that this variation may stem from individual differences in how people interpret and make sense of gratitude—a phenomenon we conceptualize as a gratitude mindset. This reasoning builds on a well-established body of research showing that individuals hold domain-specific beliefs (or “mindsets”) that shape how they interpret experiences and guide subsequent behavior (Crum et al., 2013; Dweck & Yeager, 2019). For instance, mindsets about intelligence (Blackwell et al., 2007) and stress (Crum et al., 2013) have been shown to systematically influence effort, performance, and coping. Extending this perspective to the domain of gratitude, we argue that individuals similarly differ in their core beliefs about gratitude: some view gratitude as an enriching and growth-oriented resource (GIEM), whereas others perceive it as a burdensome and constraining obligation (GIIM).

**Figure 1:**
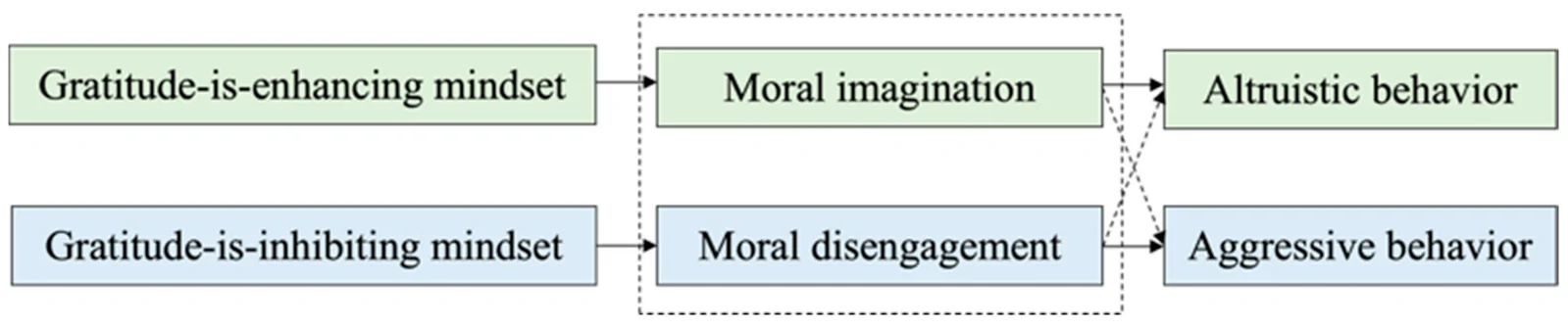
Proposed theoretical model. Note. Solid arrows represent positive relationships; dashed arrows represent negative relationships; the dashed box represents mediating variables.

Specifically, individuals with GIEM tend to view gratitude as a constructive and useful emotion (McCullough et al., 2001; Russell & Paris, 1994) that enhances social outcomes such as prosociality and interpersonal relationships (Emmons & McCullough, 2004; Ford et al., 2018). This interpretive frame, we argue, promotes moral imagination—the ability to understand contexts from multiple stakeholder perspectives, envision new possibilities, and evaluate them morally (Whitaker & Godwin, 2013; Yurtsever, 2006). Conversely, individuals with GIIM tend to perceive gratitude as a burden that threatens their autonomy or self-esteem, activating moral disengagement—a set of cognitive mechanisms that enable individuals to justify unethical behavior without experiencing guilt or self-censure (Bandura, 1999; Moore et al., 2012). Given that mindsets not only shape interpretation but also guide behavioral responses (Blackwell et al., 2007; Dweck & Yeager, 2019), we suggest that the gratitude mindset exerts its paradoxical effects on social behaviors through these two distinct moral cognitive pathways: GIEM is associated with higher altruistic behavior and lower aggressive behavior via moral imagination, whereas GIIM is associated with higher aggressive behavior and lower altruistic behavior via moral disengagement. The proposed theoretical model is illustrated in Figure 1.

Our research makes three key contributions. First, by challenging the prevailing assumption that gratitude is uniformly prosocial, we highlight the importance of individual differences in gratitude interpretation, offering a new theoretical lens for understanding when and why gratitude leads to divergent outcomes. Second, we introduce gratitude mindset as a novel construct that extends implicit theory (Dweck et al., 1995) to the domain of gratitude, broadening its explanatory scope beyond intelligence and moral character to encompass social behaviors. Third, we identify moral imagination and moral disengagement as two opposing mediating mechanisms, moving beyond prior research that has focused predominantly on motivational and self-regulatory processes (Locklear et al., 2021), thereby enriching the moral cognition perspective on gratitude-induced behaviors. Before proceeding, it is worth clarifying that our research focuses on how individuals view gratitude as a generalized interpretive framework, rather than on whether they feel grateful in a given moment or on how gratitude states are induced.

## 2. Conceptualizing Gratitude Mindset

Dweck proposed the concept of mindset, which initially included entity and incremental theories and was later renamed fixed and growth mindsets (Dweck & Yeager, 2019), aiming to explain why individuals with similar abilities exhibit differentiated adaptive behaviors when facing challenges and failures, from the perspective of motivation and personality. A mindset is defined as a mental framework or cognitive lens that selectively organizes and encodes information. Specifically, a growth mindset refers to the belief that personal attributes are malleable, whereas a fixed mindset refers to the belief that personal attributes are fixed (Dweck & Yeager, 2019). In this way, mindset orients individuals toward a distinct way of interpreting experience and guides them toward corresponding actions and responses (Crum et al., 2013). Building on the foundational entity theory and incremental theories, the construct of mindset has been expanded to numerous domains. For example, in human–AI interaction, individuals who hold an implicit belief that human mental capacities are malleable exhibit more cooperative responses to robots, whereas those who hold the implicit belief that human mental capacities are fixed display more competitive responses to robots (Dang & Liu, 2022); in the domain of intelligence, students who adopt a mindset that intelligence is malleable (rather than fixed) show improvements in their effort, motivation, and GPAs (Blackwell et al., 2007); in the stress domain, individuals who hold a stress-is-enhancing mindset demonstrate improved work performance (in both “hard” skills and “soft” skills), whereas those with a stress-is-inhibiting mindset show no such improvement (Crum et al., 2013). Unfortunately, the construct of gratitude mindset has received limited attention in the gratitude literature, despite its potential to shape not only how people respond to gratitude-eliciting situations, but also whether they actively seek out, avoid, or undermine opportunities for gratitude in their daily lives. Thus, the present study aims to explore the gratitude mindset and its downstream effects.

A gratitude mindset is proposed to act as a variable exerting an influence on the gratitude response. It refers to a cognitive framework or set of beliefs about gratitude that shapes how individuals interpret and respond to this emotion, regardless of whether they currently feel grateful. To clarify the distinction between a gratitude mindset and gratitude itself, it is first necessary to define gratitude: gratitude refers to a psychological process that occurs when individuals realize that they have successfully solved their problems due to the benefactors’ kindness rather than their own effort (McCullough et al., 2002). Furthermore, Table 1 further clarifies the distinction between a gratitude mindset and three closely related gratitude constructs. Trait gratitude refers to stable individual differences in gratitude responses (Rosenberg, 1998), whereas a gratitude mindset highlights generalized beliefs about whether experiencing gratitude is beneficial or inhibiting. State gratitude captures episodic experiences of gratitude (Emmons & McCullough, 2004), emphasizing transient reactions to specific events, whereas a gratitude mindset reflects generalized beliefs about the nature and consequences of gratitude that transcend any particular benefit-receiving episode. Expressed gratitude refers to the display and communication of an individual’s gratitude (Locklear et al., 2022), serving to reinforce relational bonds (McCullough et al., 2001) and reflecting the behavioral manifestation of gratitude experiences, whereas a gratitude mindset is the underlying interpretive framework that shapes whether and how individuals express gratitude.

Relevant theories further elaborate on gratitude’s properties: the moral affect theory (McCullough et al., 2001) highlights that gratitude motivates grateful individuals to engage in altruistic behaviors toward their benefactors and others, while encouraging benefactors to act morally in the future; the find-remind-and-bind theory (Algoe, 2012) notes that gratitude promotes relationship formation and maintenance. Together, these perspectives confirm that gratitude is an emotion-driven psychological process that encourages beneficiaries to show kindness to their benefactors and unrelated others, strengthens their social relationships, and even reinforces benefactors’ moral actions. However, these theories do not explain why gratitude may either promote or harm an individual’s social relationships, a phenomenon manifested in individuals’ altruistic or aggressive behaviors. We argue that such variation arises from differences in how individuals understand the meaning of gratitude experiences—that is, their meta-beliefs about whether gratitude is inherently enriching or burdensome. Specifically, GIEM leads people to understand gratitude as a positive, growth-oriented resource, thereby motivating them to engage in prosocial behaviors to express and amplify this gratitude. In contrast, GIIM prompts individuals to understand gratitude as a burdensome obligation or potential loss, which in turn discourages prosocial engagement or even triggers aggressive behaviors to alleviate the perceived pressure.

Importantly, gratitude mindset is also distinct from gratitude appraisal (see Table 1), consistent with the established distinction between stress mindset and stress appraisal (Crum et al., 2013). The Moral Affect Theory of Gratitude (McCullough et al., 2001) emphasizes that beneficiaries determine whether they feel grateful based on four key appraisals: (a) the value of the benefits received, (b) the cost and effort the benefactor expended to provide those benefits, (c) whether the benefactor’s effort was intentional, and (d) whether the kindness was gratuitous (rather than motivated by a role-based relationship between the benefactor and beneficiary). Thus, gratitude appraisal refers to the evaluation of received help that determines whether and how strongly one feels grateful. In contrast, a gratitude mindset refers to the belief that gratitude has either enhancing or inhibiting consequences for one’s own growth and development.

## 3. Proposed Mechanisms

The theoretical foundation of the proposition is that the gratitude mindset significantly influences how gratitude-induced behavioral responses are expressed. Prior research has established that gratitude prompts altruistic behavior and inhibits unethical conduct. For example, some experimental studies found that participants who felt gratitude induced by advertising on social media showed more donating behaviors (Baek & Yoon, 2022); a 4-year longitudinal investigation suggested that gratitude growth predicted more helping behavior over four years (Bono et al., 2019); and DeSteno et al. (2019) demonstrated that gratitude could reduce students’ cheating behaviors compared to neutrality and positive emotional states, suggesting that gratitude experiences may activate moral concerns that inhibit unethical conduct. Moreover, Sun et al. (2019) found a negative association between gratitude and aggression. Drawing on this insight, we argue that GIEM may strengthen this moral sensitivity. Specifically, individuals who hold a GIEM may be more likely to engage in altruistic behavior and less likely to engage in aggressive behavior in gratitude-eliciting situations.

On the other hand, individuals who hold a GIIM are primarily motivated to avoid gratitude because they perceive it as detrimental to their personal development. Prior research has demonstrated that gratitude can also have a dark side. For example, four experimental studies found that gratitude increased individuals’ willingness to obey commands to perform ethically questionable acts (Tong et al., 2021). In addition, Zhu et al. (2020) conducted six studies, including scenario and laboratory studies, demonstrating that gratitude increases moral violations when these violations protect their benefactors from harm. Moreover, recent research has revealed that certain gratitude-eliciting situations can evoke mixed emotional experiences (e.g., gratitude–anger and gratitude–disappointment), which are in turn associated with negative action tendencies and psychological outcomes (Oh & Tong, 2023). Parallel evidence has suggested that receiving help can sometimes motivate social undermining behavior, particularly when it threatens the recipient’s perceived status (Tai et al., 2023). These findings suggest that gratitude is not always positive; rather, it could evoke ambivalence and even hostility under certain conditions. Thus, we argue that GIIM may heighten these negative tendencies by sensitizing individuals to the potential costs of gratitude. Specifically, individuals who hold a GIIM may be more likely to engage in aggressive behavior and less likely to engage in altruistic behavior. We propose the following hypothesis:

**Hypothesis** **1.** *GIEM is (1) positively related to altruistic behavior and (2) negatively related to aggressive behavior*.

**Hypothesis** **2.** *GIIM is (1) positively related to aggressive behavior and (2) negatively related to altruistic behavior*.

The theoretical underpinning of the proposal that gratitude mindset alters personal altruistic or aggressive behavior lies in the fact that different gratitude mindsets will be associated with different moral processes. Moral imagination refers to a unique form of moral reasoning that entails understanding diverse perspectives, imagining novel possibilities, and engaging in the moral appraisal of those possibilities (Whitaker & Godwin, 2013). In other words, moral imagination enables individuals to distance themselves from their roles, creatively reframe experiences, and generate new moral alternatives (Yurtsever, 2006). Similarly, individuals who hold a GIEM always distance themselves from their own efforts and psychological states to consider the reasons for their success in solving problems from different stakeholder perspectives (i.e., themselves and helpers). Consequently, an individual who holds a GIEM will be more likely to adopt a moral imagination strategy. Moral imagination represents humans’ seeking to behave ethically and avoid negative social behaviors. Specifically, prior researchers have conducted experiments that support the proposal that moral imagination increases prosocial decision-making (Gaesser et al., 2018). Moreover, since imagination inflation has a positive effect on events related to imagination happening (Carroll, 1978; Weiler et al., 2010), we argue that moral imagination has a positive effect on altruistic behavior. Importantly, this proposition has received empirical support, as moral imagination has been linked to positive social behaviors (Pless et al., 2021). Conversely, we expected that moral imagination is negatively related to aggressive behavior. Because moral imagination focuses on the positive effects of altruistic behavior, it is less likely to harm others.

Conversely, individuals who hold a GIIM successfully avoid or refuse the gratitude mechanism and end up treating gratitude as a destructive and negative emotion, which may be hyperactivated and lead to moral disengagement (de Hooge et al., 2011). Social cognitive theory suggests that moral disengagement is related to inhibitory mechanisms (Bandura, 1999). Specifically, individuals redefine their moral standards and deactivate self-regulatory mechanisms, which enable them to be free from the consequences of aggressive behaviors (Bandura, 1999; Bowen & Blackmon, 2003). Meanwhile, we posit that moral disengagement is positively associated with aggressive behavior because it reduces prosociality and anticipatory self-censure while promoting cognitive and affective reactions conducive to aggression (Bandura et al., 1996). Similarly, a meta-analytic investigation found that moral disengagement is significantly associated with aggressive behavior (Gini et al., 2014). These findings highlighted that moral disengagement was likely to shape aggressive behavior. Thus, those with a GIIM may be more likely to adopt disengagement strategies rather than engage in moral imagination, which may in turn increase aggressive behavior and reduce altruistic behavior. In summary, we propose the following hypothesis:

**Hypothesis** **3.** *GIEM has a (1) positive indirect effect on altruistic behavior and (2) a negative indirect effect on aggressive behavior, mediated by moral imagination*.

**Hypothesis** **4.** *GIIM has a (1) positive indirect effect on aggressive behavior and (2) a negative indirect effect on altruistic behavior, mediated by moral disengagement*.

## 4. Present Research

To test the proposal that a gratitude mindset is a meaningful construct in determining personal altruistic and aggressive behavior and to explore the mechanism, we conducted two studies. Study 1 demonstrated the differential effects of GIEM and GIIM on altruistic versus aggressive behaviors. Study 2 examined the proposed mechanisms linking a gratitude mindset with altruistic and aggressive behavior. Specifically, it did so by measuring the mediating effects of moral imagination on the relationship between GIEM and altruistic and aggressive behavior, and by measuring the mediating effects of moral disengagement on the relationship between GIIM and aggressive and altruistic behavior.

## 5. Study 1

### 5.1. Samples and Procedure

We recruited 221 undergraduates from a university in China. In this experimental sample, 55.2% of the participants identified as male; the participants’ mean age was 19.54 years (*SD* = 1.58). Using G*power 3.1 software, 64 participants for each group were sufficient to detect a medium effect size (d = 0.5) with two groups, an alpha significance criterion of 0.05 (two-tailed), and statistical power = 0.80. All participants provided informed consent. Participants were told that participation was voluntary, responses were confidential, and that the data were for research only. The ethical committee at the university from which the participants hailed granted approval for the study (approval no. 202406-10a).

**Figure 2:**
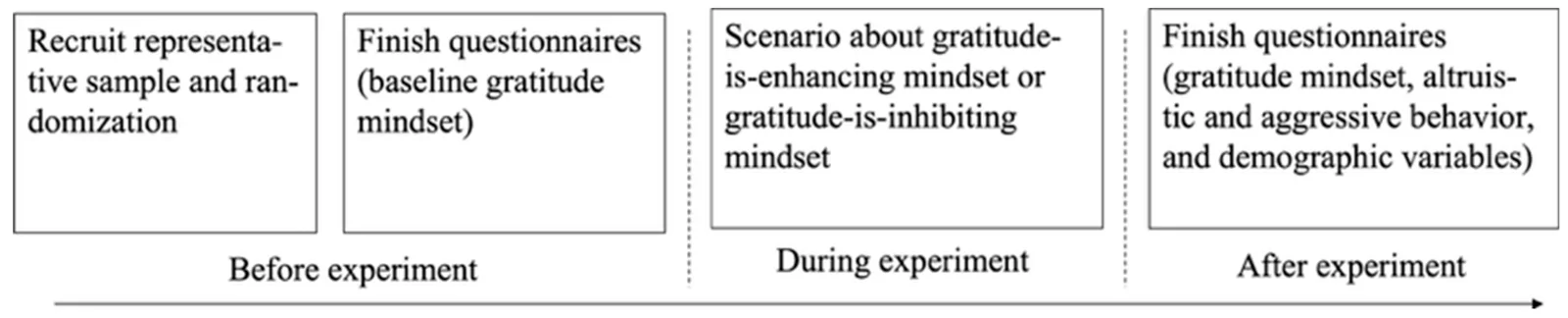
Experimental procedure of Study 1.

To test the effects of a gratitude mindset (including GIEM and GIIM) on altruistic and aggressive behavior, we conducted a between-subject design followed by Hoyt et al. (2023). The participants voluntarily applied to participate in this experiment, and they were randomly assigned to one of two conditions: GIEM condition or GIIM condition. Participants first completed a series of questionnaires assessing their baseline gratitude mindset to establish a pre-test reference for comparing changes in gratitude mindset after the experimental manipulation. We adopted standard procedures to manipulate the participants’ mindset (e.g., Hoyt et al., 2018). In the GIEM condition (*N* = 114), participants read a Gratitude Today type article to encourage them to view gratitude as a benefit that enhances social behaviors. In the GIIM condition (*N* = 107), participants read a similar article about gratitude inhibiting social behaviors. Finally, participants rated the current gratitude mindset, altruistic behavior, aggressive behavior, and demographics. The experimental procedure is illustrated in Figure 2.

### 5.2. Measures

Gratitude mindset^1^. We developed an 11-item scale to measure the gratitude mindset. GIEM includes six items such as “Gratitude brings many positive effects and should be actively utilized” (Cronbach’s α = 0.98). GIIM includes five items such as “Gratitude has many negative effects and should be avoided” (Cronbach’s α = 0.96). Response options ranged from one (strongly disagree) to six (strongly agree). Below, we detail the scale-development process.

First, we conducted semi-structured interviews with 30 undergraduate students to capture their lay beliefs about gratitude. Based on these interviews and the existing mindset literature, we generated an initial pool of 16 items. Next, we evaluated these items for redundancy and clarity. In doing so, we employed the assistance of positive psychology and mindset faculty and doctoral students. We conducted an exploratory factor analysis (EFA) with 301 undergraduates (54.2% male; *M*_age_ = 19.83, *SD* = 1.65) from a university in China. According to the relevant suggestions on the sample size for factor analysis: a sample size of 100 to 200 is the minimum requirement for EFA (Worthington & Whittaker, 2006). Thus, our sample size was sufficient. Respondents had an average age of 19.83 (*SD* = 1.65). Before applying the EFA, we employed the KMO and Bartlett’s test. The results revealed that the KMO value was 0.92, and Bartlett’s test was significant (χ^2^ = 2059.012, *p* < 0.001), confirming sampling adequacy. The scree plot and eigenvalues (>1.0) supported a clear two-factor structure, corresponding to GIEM and GIIM. The two factors accounted for 67.65% of the total variance. All 11 items loaded strongly on their respective factors (loadings > 0.50) with no significant cross-loadings (see Appendix A).

We conducted a confirmatory factor analysis (CFA) with 263 undergraduates (57.8% male; *M*_age_ = 20.93, *SD* = 1.49) from a university in China. The results provide support for the factor structure of the gratitude mindset measure. The two-factor model revealed a good fit (χ^2^/df = 2.13, CFI = 0.96, TLI = 0.95, SRMR = 0.04) and an acceptable fit (RMSEA = 0.07) to the data (Ellen et al., 2022). The CR values for all constructs were above 0.70, and the AVE values were 0.56 and 0.51, respectively (see Appendix B). Furthermore, to examine whether GIEM and GIIM represent two distinct dimensions or merely a single bipolar continuum, we conducted a series of CFAs comparing three competing measurement models (see Appendix B).

Altruistic behavior. Altruistic behavior was measured using the 22-item Chinese version of the scale from Zhou et al. (2023). Participants were asked, “I do my best when others ask for help”. Response options ranged from one (strongly disagree) to seven (strongly agree). The Cronbach’s α for this scale was 0.97.

Aggressive behavior. Aggressive behavior was measured using the 20-item Chinese version of the Buss–Perry Aggression scale (Buss & Perry, 1992), as revised by Fang (2016). The validated scale includes three subscales that reflect the composition of aggressive behavior: verbal aggression, anger, and hostility. The items (e.g., “I oppose friends directly when I disagree with their opinions.”) were rated on a scale ranging from one (strongly disagree) to seven (strongly agree). The Cronbach’s α for this scale was 0.95.

### 5.3. Analytic Strategy

Following previous studies, we conducted SPSS 27.0 for statistical analysis. Specifically, we performed a paired *t*-test and two-sample *t*-test for the manipulation check and to examine the hypothesized direct effects.

### 5.4. Results

#### 5.4.1. Manipulation Check

The paired *t*-test and two-sample *t*-test results demonstrated the effectiveness of gratitude mindset manipulations (see Table 2 and Table 3). There was no difference between the two conditions on the baseline GIEM (t (219) = 1.89, M = 4.94 vs. 4.75, *p* = 0.061, 95% CI = [−0.01, 0.38]) and GIIM (t (219) = 1.41, M = 2.27 vs. 2.43, *p* = 0.161, 95% CI = [−0.37, 0.06]). Compared to the baseline, participants in the GIEM condition reported significantly higher GIEM (t (113) = 9.11, M = 4.94 vs. 5.47, *p* < 0.001, 95% CI = [0.41, 0.64], d = 0.85), whereas participants in the GIIM condition reported significantly higher GIIM (t (106) = 14.83, M = 2.43 vs. 4.46, *p* < 0.001, 95% CI = [1.76, 2.31], d = 1.43). Meanwhile, compared to participants in the GIIM condition, participants in the GIEM condition reported significantly higher GIEM (t (219) = 24.89, M = 2.72 vs. 5.47, *p* < 0.001, d = 3.34, 95% CI = [2.53, 2.97]); compared to participants in the GIEM condition, participants in the GIIM condition reported significantly higher GIIM (t (219) = 22.79, M = 1.74 vs. 4.46, *p* < 0.001, d = 3.06, 95% CI = [2.49, 2.96])^2^.

#### 5.4.2. Hypotheses Testing

The two-sample *t*-test results demonstrated the effect of a gratitude mindset (including GIEM and GIIM) on altruistic and aggressive behavior. Specifically, compared to participants in the GIIM condition, participants in the GIEM condition reported significantly higher altruistic behavior (t (219) = 22.64, M = 3.47 vs. 5.58, *p* < 0.001, d = 3.05, 95% CI = [1.92, 2.29]); compared to participants in the GIEM condition, participants in the GIIM condition reported significantly higher aggressive behavior (t (219) = 13.59, M = 2.26 vs. 3.99, *p* < 0.001, d = 1.83, 95% CI = [1.47, 1.97]).

### 5.5. Discussion

Study 1 aimed to test the effects of GIEM (vs. GIIM) on altruistic behavior (vs. aggressive behavior). However, both the relationships between GIEM and aggressive behavior and that between GIIM and altruistic behavior remained unclear. We did not explore the psychological mechanism of the above relationship. Thus, Study 2 applied questionnaires to examine the double mediation model to understand how and why a gratitude mindset triggers altruistic and aggressive behavior.

## 6. Study 2

### 6.1. Samples and Procedure

The required sample size was calculated using G*power 3.1 software, with the effect size set to *f*^2^ = 0.15, statistical power = 0.80, and alpha = 0.05. Under these parameters, a sample size of 114 was required. We recruited 462 undergraduate students from two public courses at two universities in China. Upon completion of the online survey development, we disseminated the questionnaire links to participants through the WeChat platform. Prior to their involvement, all participants provided informed consent and were free to withdraw from the study at any time, at their discretion. Participation was voluntary and their responses were confidential. The ethical committee at the university from which the participants hailed granted approval for the study (approval no. 202406-10a).

To minimize common method variance (CMV), we measured the independent, mediating, and dependent variables by collecting data at three time points. Specifically, participants completed a six-week questionnaire survey at one-week intervals. A one-week interval was chosen to balance the need for temporal separation (to reduce common method variance) while minimizing the risk of attrition and recall bias. At Time 1, participants provided demographic information and gratitude mindset. At Time 2, they reported moral imagination and moral disengagement. At Time 3, participants reported altruistic behavior and aggressive behavior. We used identification codes for students’ responses in the three-wave survey to ensure confidentiality. Since there may be phenomena such as students’ absence or asking for leave in public courses, we received 549 completed responses at Time 1, 501 at Time 2, and 462 at Time 3, yielding a matched sample of 462 participants. In this final sample, 66.5% of the students were identified as male; the participants had an average age of 18.40 (*SD* = 0.89). Gender was coded as 0 = female and 1 = male. We also conducted attrition analyses. The results revealed no significant differences between the attrition samples and the final samples on GIEM (*t* = 0.18, *p* = 0.86), GIIM (*t* = 1.45, *p* = 0.15), gender (*t* = 1.09, *p* = 0.28), and age (*t* = 0.96, *p* = 0.34). Furthermore, we conducted a binary logistic regression analysis to examine whether these baseline characteristics systematically predicted participant attrition. The overall model was not significant, χ^2^ (4) = 5.49, *p* = 0.24. None of the predictors significantly predicted attrition, including gender (B = −0.29, *p* = 0.27), age (B = −0.09, *p* = 0.31), GIEM (B = −0.21, *p* = 0.23), and GIIM (B = −0.32, *p* = 0.08).

### 6.2. Measures

Moral imagination and moral disengagement scales were translated and culturally adapted following the translation-back-translation procedure (Brislin, 1970). Specifically, each scale was independently translated into Chinese by two bilingual translators; a consensus version was then reached and subsequently back-translated into English by two independent translators. The research team then compared the original and back-translated versions, resolving any semantic and conceptual discrepancies to ensure equivalence.

Gratitude mindset. Gratitude mindset was measured as in Study 1. The Cronbach’s α for GIEM and GIIM subscales was 0.89 and 0.79, respectively.

Moral imagination. Moral imagination was measured with an eight-item scale from Yurtsever (2006). The items (e.g., “I like to imagine the consequences of my behavior that affect others.”) are rated on a scale ranging from one (strongly disagree) to five (strongly agree). The Cronbach’s α for this scale was 0.84.

Moral disengagement. Moral disengagement was measured with an eight-item scale from Moore et al. (2012). The validated scale includes eight subscales that reflect the composition of moral disengagement: moral justification, euphemistic labelling, advantageous comparison, displacement of responsibility, diffusion of responsibility, distortion of consequences, dehumanization, and attribution of blame. The items (e.g., “It is okay to spread rumors to defend those you care about.”) are rated on a scale ranging from one (strongly disagree) to seven (strongly agree). The Cronbach’s α for this scale was 0.78.

Altruistic behavior. Altruistic behavior was measured as in Study 1. The Cronbach’s α for this scale was 0.87.

Aggressive behavior. Aggressive behavior was measured as in Study 1. The Cronbach’s α for this scale was 0.93.

Control variables. Since altruistic or aggressive behavior may vary by gender and age (Esteban & Tabernero, 2011; Hernández Blasi, 2023), we controlled for gender and age throughout our analyses. We procured demographic information regarding the participants’ gender and age.

### 6.3. Analytic Strategy

Following previous studies, we conducted SPSS 27.0 and Mplus 8.3 for statistical analysis. First, we tested common method bias and evaluated discriminant validity for all variables, employing SPSS 27.0 and Mplus 8.3. Second, we performed descriptive statistics and correlation analysis using SPSS 27.0. Finally, we analyzed the data using hierarchical regression and the PROCESS macro for SPSS 27.0, which allowed us to test the direct effects and the mediation effects hypothesized in our model.

### 6.4. Results

#### 6.4.1. Common Method Bias Test

Following Podsakoff et al. (2003), we employed Harman’s single-factor test and the unmeasured latent method construct (ULMC) to examine common method bias. The results revealed thirteen factors with eigenvalues greater than 1, and the first factor’s variance was 24.16% (<40%). After introducing a method factor to the six-factor CFA model, the model fit indices (χ^2^/df = 1.82, CFI = 0.90, TLI = 0.88, RMSEA = 0.04) did not show substantial improvement over the baseline model (χ^2^/df = 1.72, CFI = 0.91, TLI = 0.90, RMSEA = 0.04; ΔCFI = −0.01, ΔTLI = −0.02, ΔRMSEA = 0.00), indicating that common method bias was not a severe problem in this study.

#### 6.4.2. Descriptive Analysis and Correlations

The Pearson correlation between descriptive statistics and related variables is demonstrated in Table 4. The results showed that GIEM was significantly and positively associated with moral imagination (*r* = 0.25, *p* < 0.001) and altruistic behavior (*r* = 0.44, *p* < 0.001), and negatively related to moral disengagement (r = −0.21, *p* < 0.001) and aggressive behavior (*r* = −0.23, *p* < 0.001). In contrast, GIIM was significantly and positively associated with moral disengagement (*r* = 0.21, *p* < 0.001) and aggressive behavior (*r* = 0.35, *p* < 0.001) and negatively related to moral imagination (*r* = −0.27, *p* < 0.001) and altruistic behavior (*r* = −0.42, *p* < 0.001). These results provided initial evidence for our proposal. Furthermore, gender (0 = female, 1 = male) was positively associated with moral disengagement (*r* = 0.19, *p* < 0.001); age was associated with GIEM (*r* = −0.22, *p* < 0.001), GIIM (*r* = 0.18, *p* < 0.001), moral imagination (*r* = −0.10, *p* < 0.05), and altruistic behavior (*r* = −0.13, *p* < 0.01). Subsequent regression analysis will use these variables as control variables.

#### 6.4.3. Hypotheses Testing

Regression analysis. We conducted linear regression analyses to examine the direct effects of GIEM on altruistic (see Model 2 in Table 5) and aggressive behavior (see Model 2 in Table 6) and GIIM on altruistic (see Model 4 in Table 5) and aggressive behavior (see Model 4 in Table 6). After controlling for gender and age, the results showed that GIEM was positively associated with altruistic behavior (*β* = 0.43, *p* < 0.001) and negatively associated with aggressive behavior (*β* = −0.24, *p* < 0.001). GIIM, on the other hand, was positively associated with aggressive behavior (*β* = 0.36, *p* < 0.001) and negatively associated with altruistic behavior (*β* = −0.41, *p* < 0.001). These results supported H1 and H2.

Mediation effects. Here, we tested the mediation effect by applying Model 4 (controlling for gender and age) of the SPSS 27.0 macro plugin PROCESS v4.0. We estimated the total effects, direct effects, indirect effects, and their 95% bias-corrected CIs using bootstrapping with 5000 samples (see Table 7). The results showed that the direct effect of GIEM on altruistic behavior (*β* = 0.27, *SE* = 0.03, 95% CI = [0.21, 0.33]) and aggressive behavior (*β* = −0.17, *SE* = 0.05, 95% CI = [−0.28, −0.07]) were significant. Furthermore, moral imagination significantly mediated the positive relationship between GIEM and altruistic behavior (*β* = 0.06, *SE* = 0.02, 95% CI = [0.03, 0.10]). Similarly, moral imagination significantly mediated the negative relationship between GIEM and aggressive behavior (*β* = −0.10, *SE* = 0.02, 95% CI = [−0.15, −0.05]). Hence, moral imagination partially mediated the relationships between GIEM and both altruistic and aggressive behavior.

Subsequently, the relationship between GIIM and social behavior (i.e., altruistic and aggressive behavior) via moral disengagement was also investigated in great detail (see Table 7). Specifically, the results showed that the direct effect of GIIM on altruistic behavior (*β* = −0.28, *SE* = 0.03, 95% CI = [−0.34, −0.21]) and aggressive behavior (*β* = 0.33, *SE* = 0.05, 95% CI = [0.24, 0.43]) was significant. Furthermore, moral disengagement significantly mediated the positive relationship between GIIM and aggressive behavior (*β* = 0.07, *SE* = 0.02, 95% CI = [0.04, 0.12]). Similarly, moral disengagement significantly mediated the negative relationship between GIIM and altruistic behavior (*β* = −0.04, *SE* = 0.01, 95% CI = [−0.08, −0.02]). Hence, moral disengagement partially mediated the relationships between GIIM and both altruistic and aggressive behavior. Our results supported H3 and H4.

### 6.5. Discussion

In Study 2, we replicated the results of Study 1, which showed that GIEM was positively related to altruistic behavior and negatively related to aggressive behavior, while GIIM was positively related to aggressive behavior and negatively related to altruistic behavior. Furthermore, GIEM was prospectively associated with altruistic behavior and negatively associated with aggressive behavior via moral imagination, whereas GIIM was prospectively associated with aggressive behavior and negatively associated with altruistic behavior via moral disengagement.

## 7. General Discussion

We strived to explore the association between a gratitude mindset and distinct types of social behaviors (altruistic and aggressive behavior). The findings across two studies provide convergent support for the paradoxical effect of a gratitude mindset on altruistic and aggressive behavior. To begin with, Study 1 indicated that participants in the GIEM condition reported higher altruistic behavior and lower aggressive behavior, while those in the GIIM condition reported higher aggressive behavior and lower altruistic behavior. Study 2 extended the findings of Study 1 and provided preliminary insights into the potential mechanisms underlying the links between gratitude mindset, moral imagination/moral disengagement, and altruistic behavior/aggressive behavior. As mentioned previously, moral imagination mediated the positive relationship between GIEM and altruistic behavior, and also mediated the negative relationship between GIEM and aggressive behavior. In contrast, GIIM exacerbated aggressive behavior while suppressing altruistic behavior through the mechanism of moral disengagement. Given the three-wave design, these mediation effects reflect prospective associations rather than causal inferences.

### 7.1. Implications

Our research has important theoretical implications in several ways. First, this research enriches the literature on implicit theory by revealing that mindsets may also matter in the domain of gratitude. Previous research highlighted the responses to gratitude. Our work advances a new construct of gratitude mindset (i.e., GIEM and GIIM), which not only broadens implicit theory but also lays the groundwork for research on gratitude-induced behavioral outcomes. Extensive research on implicit theory has investigated mindsets about intelligence and personality and their impacts on academic performance (Yeager et al., 2019) and social development (Yeager et al., 2013). However, less is known about the impact of implicit theory on interpersonal reciprocity. Our work extends implicit theory by linking gratitude mindset to social behaviors, tightening the connection between gratitude mindset and altruistic and aggressive behavior. Meanwhile, our work answers the call from Dweck (2024) to apply implicit theory to a wider variety of fields.

Second, our findings provide a novel perspective on understanding gratitude-induced behaviors and highlight the importance of individual differences in beliefs about gratitude. Although a large amount of research has shown that gratitude is a positive emotion (McCullough & Tsang, 2004) and produces a wealth of benefits, such as facilitating prosociality (Bono et al., 2019) and donating behaviors (Baek & Yoon, 2022), our work demonstrates that gratitude mindset plays a critical role in shaping contradictory social behaviors. Specifically, individuals who hold GIEM exhibit more altruistic behavior and less aggressive behavior, whereas those with GIIM display more aggressive behavior and less altruistic behavior. Hence, our findings contribute to broadening theories of gratitude by showing how different beliefs about gratitude experiences translate into distinct behavioral outcomes.

Finally, this research incorporates moral imagination and moral disengagement as two opposing mechanisms from a moral cognition lens, expanding our understanding of the relationship between gratitude mindset and social behaviors. In fact, prior research has exhibited the following inconsistencies or gaps in two ways. On the one hand, while much of the literature has shown that gratitude increases positive social behaviors or decreases negative social behaviors, other research has highlighted that receiving help can often trigger social undermining through status threat and envy (Tai et al., 2023). On the other hand, although research has revealed some mediating mechanisms such as prosocial motivation (Bartlett & DeSteno, 2006), relationship closeness (Algoe, 2012), and self-control resources (Bono et al., 2019), little research has considered moral cognition as a mediating mechanism underlying the relationship between gratitude and social behaviors. In doing so, in order to fill the gap and reconcile the contradictory views stated above, we integrate the dual property of gratitude mindset and distinct types of social behaviors by identifying moral cognition as the mediating mechanism (i.e., moral imagination and moral disengagement), which provides a holistic understanding of the double-edged effects of a gratitude mindset on altruistic and aggressive behavior.

This research also has practical implications. On the one hand, we recommend that educators and managers implement tailored interventions to strengthen individuals’ GIEM and weaken their GIIM. This research suggests that educators and managers incorporate topics such as implicit theory and a growth mindset culture into their training courses—specifically through gratitude-related scenarios (e.g., analyzing how these cognitions shape responses to others’ kindness)—to cultivate individuals’ GIEM. These courses encompass case discussion, exchange meetings, and workshops (e.g., practice using incremental theory in social interactions, comprehend the principle of brain plasticity, Yeager et al., 2013) and are utilized with flexibility to cultivate people’s GIEM, thereby promoting altruistic behavior. On the other hand, our work suggests that educators and managers could focus on shaping individuals’ gratitude-related moral cognition through experiential and interactive approaches. Our findings place value on moral cognition, that is, GIEM-induced moral imagination promotes altruistic behavior, whereas GIIM-induced moral disengagement heightens aggressive behavior. Thus, with reference to moral imagination intervention, we recommend multifaceted curricula—enhancing moral emotion, moral perception, ethical reasoning, and critical reflection (Abowitz, 2007; Lederach, 2005). Meanwhile, we suggest that moral disengagement mechanisms be reduced through interventions such as socio-analytic thinking (D’Errico et al., 2024) and skill training (e.g., cognitive restructuring and mindfulness; Hofmann et al., 2012).

### 7.2. Limitations and Future Research

The current study also has some limitations. First, our research focused on the consequences of a gratitude mindset (i.e., its links to social behavior), leaving its antecedent formation mechanisms (e.g., developmental and cultural origins) and situational moderators (e.g., social norms) unclear. Future research could adopt longitudinal designs to track the development trajectory of gratitude mindsets across key life stages, helping clarify when and how they form and evolve. Second, we initially included social desirability as a control variable using a brief unvalidated scale. However, due to concerns about the psychometric properties of this brief measure (Fisher et al., 2016), we excluded it from the final analyses. Reassuringly, re-running the core models without this covariate yielded virtually identical results, suggesting that our conclusions are robust^3^. Nevertheless, future research should use validated social desirability measures to more rigorously rule out response bias.

Third, Study 1 has three key limitations. (a) Without a neutral control condition, we captured only the relative difference between GIEM and GIIM, not their effects relative to a baseline (Stark et al., 2026). Future research could add a neutral control group. (b) Our experiment used only a single reading-induced manipulation paradigm. Future research could employ alternative manipulations, such as praise (Mueller & Dweck, 1998) or recall tasks. (c) Our findings relied solely on self-reported without behavioral measures. Future research could incorporate behavioral paradigms to capture actual altruistic (e.g., dictator game, Eckel & Grossman, 1996; public goods game, Fehr & Rockenbach, 2004) and aggressive behaviors (e.g., Voodoo doll task, DeWall et al., 2013; Virtual Reality task, Lobbestael & Cima, 2021), strengthening the validity and generalizability of the findings. These behavioral tasks could be administered after gratitude mindset manipulation to assess behavioral outcomes in controlled social interaction contexts.

Furthermore, we were unable to empirically establish discriminant validity between a gratitude mindset and theoretically adjacent constructs (e.g., gratitude, indebtedness, and emotion beliefs). Future research should incorporate validated measures of these constructs to further clarify the distinctiveness of GIEM and GIIM. Additionally, our findings were obtained in a Chinese cultural context, whereas gratitude is traditionally viewed as a moral virtue reinforcing personal and interpersonal relationships (Harrison et al., 2025). This cultural framing may give gratitude a dual nature that aligns with our GIEM/GIIM distinction. Yet similar interpretive processes may emerge in other cultural contexts (Parker et al., 2017), suggesting cross-cultural relevance. Future research could test this generalizability in more diverse populations.

Finally, to explain the paradoxical effects of gratitude, we examined the mechanism by which GIEM enhances positive social behaviors via moral imagination, while GIIM promotes negative social behaviors through moral disengagement. Previous research suggests that gratitude enables individuals to be less likely to adopt moral disengagement (Sergeant & Mongrain, 2011; Yang et al., 2020). In other words, gratitude may have interactive effects within different moral cognitive processes (moral imagination and moral disengagement). Future research could investigate the cross-effects between the two types of gratitude mindset and the two mediating variables.

## 8. Conclusions

By integrating gratitude literature, implicit theory on gratitude and theory related to moral cognition (i.e., moral imagination and moral disengagement), we offer a fresh and nuanced look into the relationship between gratitude mindset and social behaviors. The findings confirmed that a GIEM is positively associated with altruistic behavior, and mediated by moral imagination, negatively associated with aggressive behavior. Furthermore, a GIIM is positively related to aggressive behavior, and mediated by moral disengagement, negatively associated with altruistic behavior. To our knowledge, this might be the first study to propose a gratitude mindset and empirically test how individuals with different gratitude mindsets exhibit altruistic and aggressive behavior. Given a relative dearth of empirical work investigating the paradoxical effects of gratitude mindset on social behavior, we expect our findings to contribute to spark future research endeavors on the complex impacts and crucial implications of a gratitude mindset.

## Figures and Tables

**Table 1:** Conceptual comparison of gratitude constructs.

Concept	Definition	Key Characteristics
Trait gratitude	Stable individual differences in gratitude responses	Emotional disposition
State gratitude	Captures episodic experiences of gratitude	Emotional state
Expressed gratitude	The display and communication of an individual’s gratitude	Behavioral manifestation
Gratitude appraisal	The evaluation of received help that determines whether and how strongly one feels grateful	Emotional appraisal
Gratitude mindset	Beliefs about gratitude that shape how individuals interpret and respond to this emotion	Beliefs

**Table 2:** Manipulation check results for Study 1 (two-sample *t*-test).

	M (*SD*)		t	*p*	Cohen’s d	95% CI
	GIEM Condition (*n* = 114)	GIIM Condition (*n* = 107)				
Baseline GIEM	4.94 (0.76)	4.75 (0.70)	1.89	*p* = 0.061	–	[−0.01, 0.38]
Baseline GIIM	2.27 (0.82)	2.43 (0.80)	1.41	*p* = 0.161	–	[−0.37, 0.06]
Post GIEM	5.47 (0.63)	2.72 (0.98)	24.89	*p* < 0.001	3.34	[2.53, 2.97]
Post GIIM	1.74 (0.79)	4.46 (0.98)	22.79	*p* < 0.001	3.06	[2.49, 2.96]

Note. Cohen’s d was not calculated for non-significant baseline comparisons.

**Table 3:** Manipulation check results for Study 1 (paired *t*-test).

Condition	Variable	M (*SD*)	t	*p*	Cohen’s d	95% CI
GIEM condition (*n* = 114)	Baseline GIEM	4.94 (0.76)	9.11	*p* < 0.001	0.85	[0.41, 0.64]
	Post GIEM	5.47 (0.63)				
GIIM condition (*n* = 107)	Baseline GIIM	2.43 (0.80)	14.83	*p* < 0.001	1.43	[1.76, 2.31]
	Post GIIM	4.46 (0.98)				

**Table 4:** Study 2 descriptive statistics and correlations.

*Variables*	*M*	*SD*	*1*	*2*	*3*	*4*	*5*	*6*	*7*	*8*
1 Gender (Time 1)	0.66	0.47	1							
2 Age (Time 1)	18.40	0.89	0.06	1						
3 GIEM (Time 1)	4.69	0.80	0.02	−0.22 ***	1					
4 GIIM (Time 1)	2.30	0.79	−0.05	0.18 ***	−0.56 ***	1				
5 Moral imagination (Time 2)	3.72	0.55	0.002	−0.10 *	0.25 ***	−0.27 ***	***1***			
6 Moral disengagement (Time 2)	2.93	0.92	0.19 ***	0.03	−0.21 ***	0.21 ***	−0.14 **	1		
7 Altruistic behavior (Time 3)	4.74	0.62	−0.05	−0.13 **	0.44 ***	−0.42 ***	0.43 ***	−0.33 ***	1	
8 Aggressive behavior (Time 3)	3.19	0.91	0.06	0.03	−0.23 ***	0.35 ***	−0.38 ***	0.36 ***	−0.61 ***	***1***

Note. This table was based on the sample (*N* = 462) from Study 2. Gender: 0 = female, 1 = male. * *p* < 0.05 (two-tailed). ** *p* < 0.01 (two-tailed). *** *p* < 0.001 (two-tailed).

**Table 5:** Hierarchical regression results of altruistic behavior (standardized coefficients).

Variables	Altruistic Behavior				
	Model 1	Model 2	Model 3	Model 4	Model 5
Control Variables					
Gender	−0.04	−0.06	−0.06	−0.07	−0.02
Age	−0.13 **	−0.03	−0.02	−0.06	−0.06
Factors					
GIEM		0.43 ***	0.35 ***		
GIIM				−0.41 ***	−0.35 ***
Moral imagination			0.34 ***		
Moral disengagement					−0.25 ***
Model parameters					
*R* ^2^	0.02	0.20	0.30	0.18	0.24
*F*	4.56 *	37.18 ***	49.18 ***	33.60 ***	35.96 ***

Note. *N* = 462. Gender: 0 = female, 1 = male. * *p* < 0.05 (two-tailed). ** *p* < 0.01 (two-tailed). *** *p* < 0.001 (two-tailed). Values are standardized regression coefficients (*β*).

**Table 6:** Hierarchical regression results of aggressive behavior (standardized coefficients).

Variables	Aggressive Behavior				
	Model 1	Model 2	Model 3	Model 4	Model 5
Control Variables					
Gender	0.05	0.06	0.06	0.08	0.02
Age	0.03	−0.03	−0.04	−0.04	−0.03
Factors					
GIEM		−0.24 ***	−0.15 ***		
GIIM				0.36 ***	0.29 ***
Moral imagination			−0.35 ***		
Moral disengagement					0.30 ***
Model parameters					
*R* ^2^	0.004	0.06	0.17	0.13	0.21
*F*	0.83	9.30 ***	23.43 ***	22.10 ***	29.82 ***

Note. *N* = 462. Gender: 0 = female, 1 = male. *** *p* < 0.001 (two-tailed). Values are standardized regression coefficients (*β*).

**Table 7:** Bootstrap significance test of mediation effects.

Model	Path	Effect	Boot SE	Boot 95% CI
GIEM → moral imagination → altruistic behavior	Total effects	0.34	0.03	[0.27, 0.40]
	Direct effects	0.27	0.03	[0.21, 0.33]
	Indirect effects	0.06	0.02	[0.03, 0.10]
GIEM → moral imagination → aggressive behavior	Total effects	−0.27	0.05	[−0.37, −0.17]
	Direct effects	−0.17	0.05	[−0.28, −0.07]
	Indirect effects	−0.10	0.02	[−0.15, −0.05]
GIIM → moral disengagement → altruistic behavior	Total effects	−0.32	0.03	[−0.39, −0.25]
	Direct effects	−0.28	0.03	[−0.34, −0.21]
	Indirect effects	−0.04	0.01	[−0.08, −0.02]
GIIM → moral disengagement → aggressive behavior	Total effects	0.41	0.05	[0.31, 0.51]
	Direct effects	0.33	0.05	[0.24, 0.43]
	Indirect effects	0.07	0.02	[0.04, 0.12]

Note. *N* = 462. Covariates: gender (0 = female, 1 = male) and age. Values are standardized regression coefficients (*β*).

## Data Availability

The raw data supporting the conclusions of this article will be made available by the authors upon request.

## Notes

1 Notably, it is worth distinguishing GIIM from the related construct of indebtedness. Although both involve negative orientations toward gratitude-related experiences, they differ in three critical ways. First, indebtedness is a discrete negative emotion triggered by receiving a favor and is conceptually opposed to gratitude (Peng et al., 2018), whereas GIIM is an implicit cognitive framework or belief system about gratitude. Second, indebtedness motivates recipients to repay benefactors to restore equity (Greenberg, 1980), whereas GIIM predicts aggressive behavior directed toward unrelated others. Third, indebtedness measures assess post-help emotional states (e.g., feeling obligated; Peng et al., 2018), whereas GIIM measures capture general negative beliefs about gratitude (e.g., “Gratitude has many negative effects and should be avoided”). These distinctions suggest that GIIM is not merely a proxy for indebtedness but a conceptually independent construct.

2 These d values reflect relative differences between two experimental conditions (GIEM vs. GIIM) rather than absolute effects relative to a neutral baseline. The design feature should be taken into account when interpreting these effect sizes.

3 Including social desirability as a covariate in the regression models did not change the overall pattern of results. Specifically, after controlling for gender, age, and social desirability, GIEM was positively associated with altruistic behavior (*β* = 0.40, *p* < 0.001) and negatively associated with aggressive behavior (*β* = −0.20, *p* < 0.001). Moral imagination significantly mediated the positive relationship between GIEM and altruistic behavior (*β* = 0.06, *SE* = 0.02, 95% CI = [0.03, 0.10]) and the negative relationship between GIEM and aggressive behavior (*β* = −0.09, *SE* = 0.02, 95% CI = [−0.15, −0.05]). In contrast, GIIM was positively associated with aggressive behavior (*β* = 0.27, *p* < 0.001) and negatively associated with altruistic behavior (*β* = −0.36, *p* < 0.001). Moral disengagement significantly mediated the positive relationship between GIIM and aggressive behavior (*β* = 0.05, *SE* = 0.02, 95% CI = [0.02, 0.09]) and the negative relationship between GIIM and altruistic behavior (*β* = −0.03, *SE* = 0.01, 95% CI = [−0.06, −0.01]).

## Abbreviations

GIEM	Gratitude-is-enhancing mindset
GIIM	Gratitude-is-inhibiting mindset

## Appendix A

**Table A1:** Rotated factor loadings for the two factors of the gratitude mindset scale (*N* = 301).

Items	Factors	
	GIEM	GIIM
**GIEM**		
V2	0.75	
V4	0.70	
V6	0.84	
V8	0.81	
V10	0.72	
V11	0.75	
**GIIM**		
V1		0.75
V3		0.78
V5		0.80
V7		0.77
V9		0.82

## Appendix B

### Appendix B.1

To rigorously evaluate whether the gratitude mindset comprises two distinct dimensions (GIEM and GIIM) rather than a single bipolar construct, we conducted a series of CFAs comparing three competing measurement models (see Table A2).

**Table A2:** Model fit comparison.

Model	χ^2^	df	χ^2^/df	RMSEA	SRMR	CFI	TLI
Model 1	358.60	44	8.15	0.17	0.11	0.76	0.70
Model 2	91.59	43	2.13	0.07	0.04	0.96	0.95
Model 3	159.04	35	4.54	0.12	0.33	0.90	0.85

Note. All models in this table were based on the CFA sample (*N* = 263) from Study 1. Model 1: A single bipolar gratitude mindset factor; Model 2: A correlated two-factor model of GIEM and GIIM (the hypothesized model); Model 3: A bifactor model.

The results revealed that Model 2 yielded acceptable fit (χ^2^/df = 2.13, CFI = 0.96, TLI = 0.95, RMSEA = 0.07, SRMR = 0.04) and fit significantly better than either Model 1 (χ^2^/df = 8.15, CFI = 0.76, TLI = 0.70, RMSEA = 0.17, SRMR = 0.11) or Model 3 (χ^2^/df = 4.54, CFI = 0.90, TLI = 0.85, RMSEA = 0.12, SRMR = 0.33). These findings support that GIEM and GIIM are two distinct constructs.

### Appendix B.2

**Table A3:** Correlations, AVE, and CR.

	1	2	AVE	CR
GIEM	1		0.56	0.89
GIIM	−0.47 ***	1	0.51	0.84

Note. This table was based on the CFA sample (*N* = 263) from Study 1. *** *p* < 0.001 (two-tailed).
